# Privilege, social justice and the goals of medicine: Towards a critically conscious professionalism of solidarity

**DOI:** 10.1007/s40037-022-00699-8

**Published:** 2022-03-07

**Authors:** Saleem Razack, Marco Antonio de Carvalho Filho, Gia Merlo, William Agbor-Baiyee, Janet de Groot, P. Preston Reynolds

**Affiliations:** 1 Pediatrics and Health Sciences Education, McGill University, Montreal, Canada; 2 Department of Clinical Sciences, Faculty of Veterinary Medicine, Utrecht University, Utrecht, The Netherlands; 3 New York University, New York, USA; 4 Rosalind Franklin University, North Chicago, USA; 5 Cumming School of Medicine, University of Calgary, Calgary, Canada; 6 University of Virginia, Charlottesville, VA USA

## Box 1. Illustrative cases

Case 1: Jamal, a 14-year-old Black boy who died of drowning injury, is being discussed at mortality review.

Staff 1: Gee, this is the fourth Black kid who died from drowning admitted to our unit in as many years.

Staff 2: I wonder if there is some associated factor or causal link that we may not be aware of ….

Case 2: Neville, a recent wheelchair user with myotonic dystrophy, is a 22-year-old young man. His discharge is being discussed with the interprofessional team. A joke is made that the family must be enjoying their little “vacation” from caring for Neville, which draws the smirks of team members. Outside the hospital, a team member observes Neville being carried up two flights of stairs by his elderly grandfather. She relates the story to her preceptor who says, “Don’t feel too bad. Not surprising, the waiting list for adaptations for disability can be 12–15 months.”

Case 3: Hannah, an Indigenous woman is in Emergency and films her interactions with nursing and physician staff on her smart phone, posting the recordings to Facebook in real time [1]. Many racist epithets and dismissing of symptoms can be heard on the recordings. Hannah dies in Emergency and is later diagnosed as having succumbed to COVID-19. The nearby university faculty of medicine puts out a statement denouncing the lack of professionalism of the nursing and physician team, recognizing the role of systemic racism in the events. In the statement, decanal leadership pledges to “stand in solidarity with Indigenous communities”. The statement draws the ire of Indigenous communities nation-wide. “We are sick and tired of you saying that you ‘stand in solidarity’ with us without changing anything.”

What do the cases in Box 1 have to do with medical professionalism? Case 1 addresses aspects of social justice as a core element of professionalism. Case 2 leads us to emphasize interprofessional professionalism as illustrated in essential and comprehensive discharge planning. The filmed behaviours in Case 3 are surely recognized as unprofessional, but what of the statement?

Professionalism in medicine has been defined as a social contract between society and physicians [2]. Physicians are upholders, gatekeepers and primary agents within healthcare systems, which are themselves regarded as acting benignly in patients’ best interests. Physicians also define these systems’ “truths”—efficacy, equity, and excellence. The rallying motto of this professionalism might be *noblesse oblige, *a term criticized as providing those occupying the higher station with a convenient justification for their privilege [3].

The disparities exposed by the COVID-19 pandemic, the murder of George Floyd in Minneapolis and the subsequent Black Lives Matter protests that it ignited worldwide highlight the pervasive role of societal inequities in the lives and health of marginalized people [4–7]. It is no longer possible to conceive of and teach about professionalism and healthcare systems as benignly constructed.

A new formulation of professionalism is required. There is admission that the design and logic of systems of healthcare and professional education are seminal in reproducing structures of systemic racism and discrimination. This recognition is necessary to raise up a new generation of socially conscious and professionally responsible health professionals.

We look to Brazilian educator Paulo Freire’s work for a way forward. Freire’s pedagogy arose in the social inequality that marks Latin American countries [8]. Freire viewed education as a political enterprise towards full citizenship rooted in values of social justice. Education is liberation, with its major objective being development of critical consciousness, defined as a capacity to analyze reality for its oppressive elements to change them. Liberation replaced what Freire called “banking education,” which delivers pacified individuals capable of following the rules dictated by an elite not interested in sharing power.

The concept of liberation and critical consciousness is highly relevant when applied to the medical professional relationship [9]. The liberation of patients demands that professionals support their empowerment to join the conversation about what constitutes good professionalism, relevant health outcomes, and responsive healthcare systems. Liberated professionals welcome patients’ voices in a democratic dialogue and share power to act *with* patients to transform their realities. Democratic dialogue based on mutual respect gives birth to a Professionalism of Solidarity, with critical consciousness as its major skill.

How might the three cases be analyzed through the lens of critical consciousness?

In the first case, with the noted higher drowning rate for Black children versus others, professional efficacy is rooted in the skills to notice a pattern of concern. Is noticing enough, or must action follow? A professionalism of solidarity embraces a commitment to act in partnership with communities to fulfil unmet needs, such as swimming and life-saving skills.

In the second case, the professionals model unprofessional and discriminatory language, which is all too common. Are they responsible for their ignorance of the family’s lived reality? A professionalism of solidarity emphasizes the need for understanding a patient’s social context as primary to the delivery of care.

The third case illuminates the structuring of professional self-regulation in helping to propagate systemic discrimination. It forces us to look at physicians as a group. Ownership of the profession’s role in past injustices and commitment to action in this case would be important steps of solidarity with structurally marginalized people. The competency here is attitudinal—not assuming that healthcare systems and structures are constructed benignly. A professionalism of solidarity demands we be committed to work for change for greater social justice.

The International Charter of Medical Professionalism is guided by the three principles:

1. Primacy of patient welfare;
2. Commitment to patient autonomy;
3. Principle of social justice [10].

Reformulating the charter towards a professionalism of solidarity calls for the development of structural competency in physicians, coupled with a commitment to act together in partnership with all stakeholders implicated in addressing the specific inequities, the two basic elements of critical consciousness. Structural competency has been defined as a set of analytic skills that highlight how institutional and societal structures operate to constrain individuals’ agency (i.e., ability to act) for their health and illness,[11] at all levels—hospitals, clinics, healthcare systems and the like.

Prioritizing the primacy of patient welfare, a professionalism of solidarity would invite us to note when medicine and its institutions risk being part of systems of harm and inequity, and to adjust care and recommendations with this knowledge in mind. Considering patient autonomy within relationships, a professionalism of solidarity provides the medical practitioner with the habit of mind analyze and seek to understand how individual patients’ lives and agencies are constrained by unjust structures.

A professionalism of solidarity enacted through critically conscious reflection addresses the reality of a world in which healthcare systems are key components of structural inequities. The social contract formulation has been the subject of critique[12] because it sets physicians apart from society, and it assumes relatively unconstrained agencies on the part of both parties within the contract, failing to recognize the role of unjust structures in producing behaviours viewed as “professional”.

A more apt formulation of professionalism is one of a covenant of solidarity between physicians and the societies they serve. A covenant is defined as an agreement which brings forth a relationship of mutual commitment. A professionalism that is a covenant of solidarity emphasizes the interconnectedness and mutual interdependence of all actors within it.

In addition to teaching a professionalism in which physicians use their special knowledge and technical skills to cure disease, promote quality of life and good health, which are excellent ideals, we must somehow let learners know that through the course of it all, they will be vomited on, literally or figuratively. When this happens, a professionalism of solidarity would ask that they be fully present, seeing the *whole room*, and committed to act.
